# The Hospital Officers' Association

**Published:** 1907-11-09

**Authors:** 


### THE HOSPITAL OFFICERS' ASSOCIATION.

### INTERESTING ADDRESS BY THE PRESIDENT.

The annual meeting of this association, held on November 2, was probably the most successful gathering of the kind in its history. The guest of the evening was the Right Hon. the Lord Mayor of London, who added greatly to the success of the evening by his geniality and good fellowship. Mr. Thomas Ryan, Secretary of St. Mary's Hospital, the President of the Association, was in the chair, and made a most practical and helpful speech. He assured the Lord Mayor that his presence there that evening was very welcome, and it made the members both delighted and proud to receive so distinguished and honoured a guest. He further emphasised the value of the work done in the hospital field by another guest, Mr. J. S. Wood, formerly secretary of the Chelsea Hospital for Women, and now identified with journalism as the active spirit in connection with *The Gentlewoman* newspaper. Speaking of the ladies, the President declared that their presence imparted brightness, refinement, and everything that was delightful to the occasion.

Referring to the Association, which was established in March 1902 to promote the social and professional wellbeing of hospital officers, he addressed himself particularly to junior officers who had not yet reached the highest position in their respective spheres of hospital work. He properly insisted that, to be effective for good, the work of the Association at its meetings must be pursued in a spirit of comradeship, and that the more its objects were realised the greater would be the benefits directly to the junior officers, and indirectly, but certainly, to the institution to which they were attached. The interests of the hospitals, and that of their officers, must be identical, for whatever promoted the wellbeing of the latter must tend to further the welfare of the former. "Show me your friends," says an old proverb, "and I will tell you what you are." Paraphrasing this, Mr. Ryan declared, as the result of many years' experience, "Show me your officers, and I will tell you whether your hospital is well or ill conducted."

Unfortunately this view was not appreciated or understood by all hospital committees, if any judgment of their opinion might be formed by the meagre salaries which are sometimes paid to hospital officers. There was too often a feeling that the mere routine official was best, whereas every well-conducted hospital should take care that every member of its staff was a real, live, purposeful man.

Membership of the Association conferred direct advantages of an educational character, by enabling each worker to associate with his fellows in the consideration of hospital questions, and by learning the routine business of the committee-room, and much which was helpful in many ways to a man of intelligence, who identified himself with the conduct of its affairs as a member of council, or as one of its honorary officers. The indirect advantages, too, were no less real. If the Association was to succeed, and it was succeeding, membership must confer a certain prestige and standing upon each of its members, and so aid him in the achievement of a successful career. The danger of all organisations of the kind was that, the management might fall too much into the hands of one or two men. This could be best obviated by all the members becoming actively interested in its affairs, and it rested with them whether they obtained good value for their money or not, for the greater the activity and zeal of the individual members, the greater must be the reputation attaching to such an organisation and to all who were connected with it. He referred at some length to the reorganisation of *The Hospital Gazette*, the monthly journal of the Association, the whole of the editorial work being done without payment, and special praise being due to Mr. Burleigh, who had edited it in an able and genial fashion from the outset of its career. The growth of the membership had been remarkable. In 1904 there were 95 members on the books; at the present time the number had increased to 206, of whom 146 were officers attached to London hospitals, and 60 to provincial institutions. Mr. Ryan's address made a marked impression upon those present, and from our knowledge of his administrative gifts and wide hospital experience, we are confident that, under his presidency, the Hospital Officers' Association will have a most prosperous and satisfactory year.

